# Explainable machine learning framework for predicting long-term cardiovascular disease risk among adolescents

**DOI:** 10.1038/s41598-022-25933-5

**Published:** 2022-12-19

**Authors:** Haya Salah, Sharan Srinivas

**Affiliations:** 1 Department of Industrial and Systems Engineering, University of Missouri, Columbia, MO 65211 USA; 2 Department of Marketing, University of Missouri, Columbia, MO 65211 USA; 3 Institute for Data Science and Informatics, University of Missouri, Columbia, MO 65211 USA

**Keywords:** Cardiology, Risk factors

## Abstract

Although cardiovascular disease (CVD) is the leading cause of death worldwide, over 80% of it is preventable through early intervention and lifestyle changes. Most cases of CVD are detected in adulthood, but the risk factors leading to CVD begin at a younger age. This research is the first to develop an explainable machine learning (ML)-based framework for long-term CVD risk prediction (low vs. high) among adolescents. This study uses longitudinal data from a nationally representative sample of individuals who participated in the Add Health study. A total of 14,083 participants who completed relevant survey questionnaires and health tests from adolescence to young adulthood were chosen. Four ML classifiers [decision tree (DT), random forest (RF), extreme gradient boosting (XGBoost), and deep neural networks (DNN)] and 36 adolescent predictors are used to predict adulthood CVD risk. While all ML models demonstrated good prediction capability, XGBoost achieved the best performance (AUC-ROC: 84.5% and AUC-PR: 96.9% on testing data). Besides, critical predictors of long-term CVD risk and its impact on risk prediction are obtained using an explainable technique for interpreting ML predictions. The results suggest that ML can be employed to detect adulthood CVD very early in life, and such an approach may facilitate primordial prevention and personalized intervention.

## Introduction

Cardiovascular disease (CVD) is the leading cause of death worldwide, representing 32% of all global deaths^1,2^. In 2018, coronary heart disease (CHD) was the leading cause of deaths (42.1%) attributable to CVD, followed by stroke (17.0%), high blood pressure (11.0%), heart failure (9.6%), diseases of the arteries (2.9%), and other CVD (17.4%)^3^. According to the Centers for Disease Control and Prevention (CDC), more than 200,000 deaths from heart disease and stroke each year are preventable. However, the primary challenge is that the treatment and intervention strategies used for CVD are initiated late due to several reasons, such as lack of awareness, symptoms, motivation, or misconceptions. Although CVD detection appears later in life, the risk factors leading to CVD begin in childhood as young as three years old, develop in early adulthood, and manifest into clinical disease in later stages^4,5^. Compelling research and empirical studies have identified several childhood/adolescent risk factors associated with CVD in adulthood. These include different adolescent behaviors and characteristics, such as unhealthy diet, smoking, physical inactivity, obesity, blood pressure, and lipids^6–8^. Thus, CVD risk assessments among adolescents can facilitate early intervention and primordial prevention. However, a clinical decision support tool for adolescents’ long-term CVD risk prediction does not exist.

The association between childhood/adolescent risk factors and CVD development in adulthood has been investigated extensively in the literature. Several prior works examined the impact of a single risk factor, such as adolescent body mass index (BMI) or hypertension, on CVD^9–11^. Few studies also investigated adolescent lifestyle factors (i.e., smoking, diet, and physical activity) and their association with CVD in adulthood^12–14^. For instance, Van De Laar et al. found adolescent smoking to be associated with higher arterial stiffness in adulthood^14^. Mikkilä et al. showed that a dietary pattern characterized by high consumption of rye, potatoes, butter, sausages, milk, and coffee was positively correlated with developing subclinical atherosclerosis among men^12^. On the other hand, mental health-related factors, such as stress and depression, were found to be associated with poor health outcomes, including CVD^15–17^. In addition to the traditional CVD risk factors, social determinants of health, which can be represented by socioeconomic status (SES), are significantly associated with CVD development^18^. According to previous research, four factors of SES have revealed an association with CVD in high-income countries: income level, educational attainment, employment status, and environmental factors^19,20^.

Prior research also investigated the relationship between multiple risk factors, such as biomarkers and lifestyle factors in childhood and the development of CVD later in life^13,21^. Although most of the previous research seeks to find an association between adolescent risk factors and adulthood CVD risk, some researchers developed multivariable prediction algorithms to assist clinicians in CVD risk assessment among adults^22–24^. Most of the previous risk prediction algorithms for CVD used a limited number of risk factors and assumed a linear relationship between CVD events and input predictors. On the other hand, few studies have employed machine learning (ML) models to predict CVD risk^25,26^. Nevertheless, existing association studies and prediction models have several limitations. First, most previous research focuses on the impact of a single risk factor (such as gender, total cholesterol, HDL cholesterol, systolic blood pressure, and smoking) on CVD, thereby providing limited scope for risk assessment among adolescents. Second, studies that consider the impact of more than one risk factor (such as Framingham risk-score model and ASCVD Risk Estimator Plus) on CVD use different forms of regression or multivariate analysis and assume the risk factors are related to CVD in a linear pattern. As a result, the complex synergistic interaction of risk factors is not recognized. Third, almost all the existing prediction models use factors such as age, gender, race, cholesterol, blood pressure, and diabetes status to estimate the 10-year or 30-year risk of heart disease or stroke and do not consider other behavioral and lifestyle factors as predictors. Most importantly, all existing risk prediction models are applicable only to adults above the age of 30 years and are not suitable for determining the long-term impact of unhealthy behavior in the earlier adolescent years. Finally, very little academic research is devoted to developing a predictive model that can categorize adolescents as high or low risk of CVD in adulthood. This research aims to overcome the aforementioned limitations in the literature by addressing the following questions:

(i) Can ML algorithms use risk factors pertaining to adolescents (i.e., socioeconomic, demographic, lifestyle, stressful life event, positive mood, self-image, and depressive symptoms) and predict their long-term (or adulthood) CVD risk?
(ii) Which adolescent risk factors are key predictors of adulthood CVD?
(iii) Can black-box ML models for CVD risk prediction be converted into more transparent and explainable solutions?

Specifically, this research innovates the field in three ways. First, the proposed risk prediction model applies to the adolescent population, while currently developed CVD risk calculators only apply to the adult population. Second, while the statistical expectations of the currently used CVD risk calculator limit the model’s prediction ability, the proposed risk scoring method is unconstrained, assuming all possible forms of relatedness of risk factors and incidence of CVD risk. We will develop non-parametric machine learning (ML) models, which tend to identify relationships previously masked through the use of stochastic models^27,28^. Finally, we will identify the influence and relative importance of each adolescent risk factor in predicting the adulthood CVD risk score, which, in turn, can highlight new pathways for CVD.

## Methods

**Figure 1:**
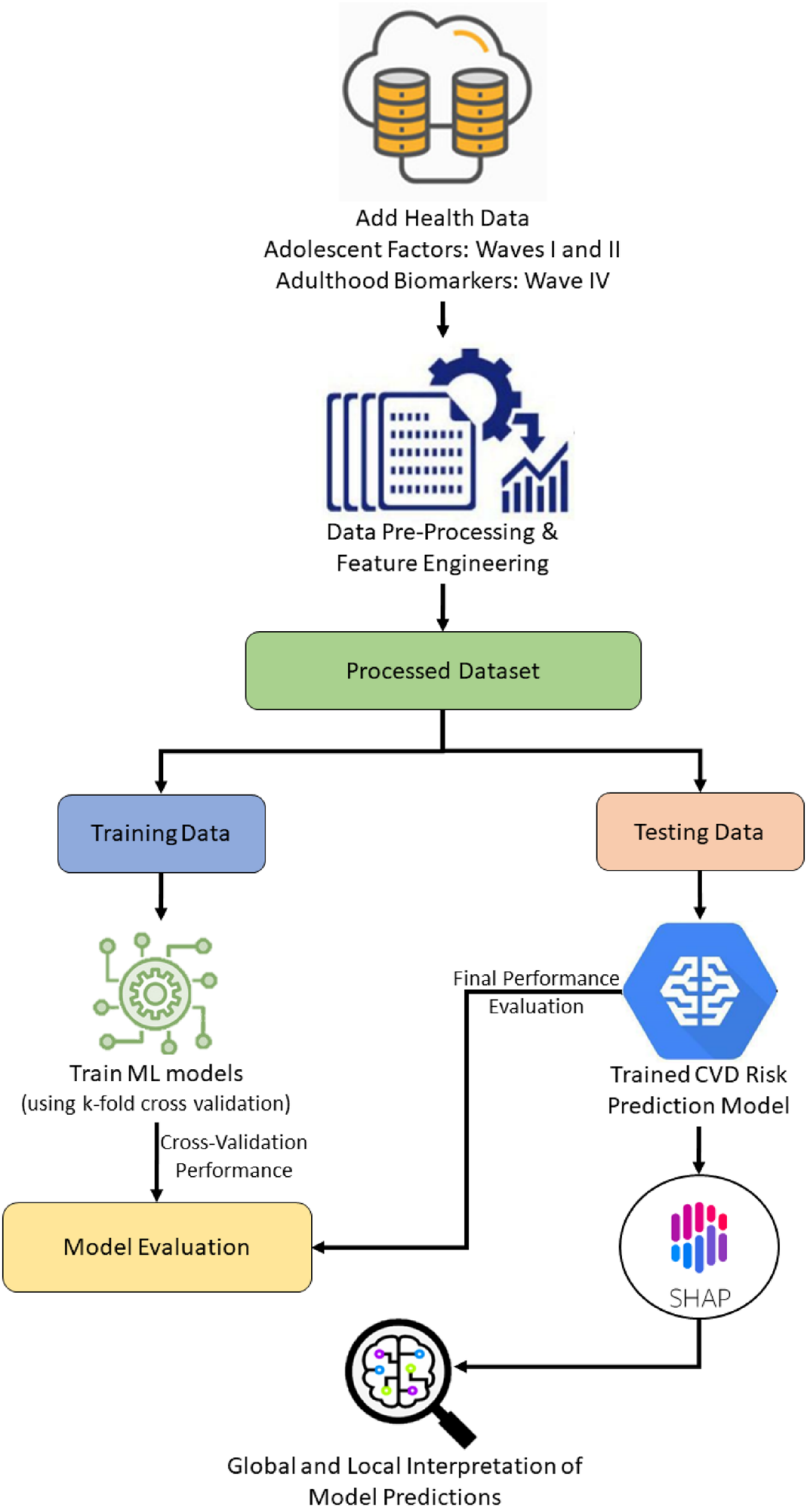
Overview of explainable machine learning framework for CVD risk prediction.

An overview of the methodology is shown in Fig. 1. We leveraged Waves I and II Add Health data to identify potential adolescent risk factors (predictors) and Wave IV data to estimate the CVD risk (outcome). The data is first pre-processed and then partitioned into training and testing subsets. The training subset is employed to train the ML models, while the testing set is used to evaluate the trained algorithm. In addition, the results from ML models are explained using the Shapley Additive exPlanations (SHAP) method.

### Data description

This study uses data from a nationally representative sample of adolescents who participated in the National Longitudinal Study of Adolescent to Adult Health (Add Health)^29^. The study followed over 20,000 individuals from adolescence to adulthood, starting with a school questionnaire and home interview for students in grades 7 through 12 from 1994 to 1995 (Wave I). The Add Health cohort was followed into young adulthood with follow-up multi-wave in-home interviews: Wave II (1996), Wave III (2001–2002), Wave IV (2007–2008). The study participants provided written informed consent for participation in all aspects of Add Health study in accordance with the University of North Carolina School of Public Health Institutional Review Board (IRB). We obtained access to the restricted-use Add Health data by completing the contractual and data use agreement. In addition, the retrospective secondary data analysis conducted in this research was approved by the University of Missouri IRB, and all methods were carried out in accordance with relevant guidelines and regulations.

In this research, we use Waves I and II (adolescent stage) data as predictors and Wave IV biomarkers (adulthood stage) for estimating the long-term CVD risk. For Wave I, the in-school questionnaire asked adolescents about their social demographics, parents’ education, occupations, self-esteem, health status, and risk behaviors. The in-home interviews included questions regarding nutrition, family composition and dynamics, substance use, and criminal activities. In addition, a parent, preferably the resident mother, was asked to complete an interviewer-assisted questionnaire on topics such as inheritable health conditions, relationships, education, employment, and income. Of all participants in Wave I, 14,738 were followed up in Wave II. The data collected in this stage was similar to Wave I, but also included more detailed nutrition information. In Wave IV, 15,701 participants from Wave I were followed into adulthood and several health-related biomarkers such as height, weight, waist circumference, and cardiovascular measurements, including systolic blood pressure, diastolic blood pressure, pulse, metabolic measures from lipids, glucose, and glycosylated hemoglobin (HbA1c), measures of inflammation and immune function were recorded. For more details about the Add Health study and design, readers can refer to Harris et al.^29^. For this research, we included all participants who were in the adolescent stage (between 10 and 19 years) during Waves I and II. Nevertheless, if a study participant was diagnosed with a heart-related disease in these waves, then that individual is excluded from our analysis.

### Data preparation

The raw data is pre-processed and prepared for predictive modeling. The predictors included both continuous and categorical variables. However, certain variables contained missing values because the participant did not know the most appropriate option for that item or refused to provide an answer. All missing values are imputed using chained equations^30^, where the distribution of unobserved values is estimated based on the observed values. In particular, if there are *M* independent variables, then the variable (e.g., ${x}_{1}$) with missing values is regressed on the other independent variables, (${x}_{2}$, ${x}_{3}$…, ${x}_{M}$), by considering only the observed values, and subsequently, the missing values in ${x}_{1}$ are estimated using the predictions from the fitted model. The procedure is repeated for each variable containing one or more missing values to obtain a complete dataset. Subsequently, each categorical variable is one-hot encoded and transformed into multiple numeric fields. The pre-processed dataset includes 14,083 complete records, which are then used for ML model development. The procedure for preparing the predictors and outcome variable is described in the following subsections.

#### Input variables (predictors) and feature engineering

Since Add Health survey questions are not specifically targeted toward CVD risk factors, many survey items are not pertinent to this research. Therefore, we selected relevant questions based on expert opinion (e.g., endocrinologist or cardiologist) and prior research findings^31–45^. Survey items from Waves I and II that reflected the following factors are selected as input variables for ML model development—sociodemographic, socioeconomic, lifestyle and health risk, stressful life events, positive well-being, and depression. While some independent variables (e.g., gender, age) can be directly obtained from Waves I and II survey questionnaires, some predictors must be inferred from one or more survey items. We rely on established survey items that are validated or employed in prior literature to infer these predictors (see Table 1). For instance, adolescents’ physical activity is obtained from the seven survey questions where individuals are asked to report how many times they are engaged in a specific activity in the last week. The responses from these questions are aggregated to create the adolescents’ total physical activity variable^33^. For sedentary behavior, the total screen time is captured based on the following questions—“How many hours a week do you watch television?”, “How many hours a week do you watch videos?” and “How many hours a week do you play video or computer games?” Participants’ responses are summed to obtain the total number of hours of screen-time per week^35^.

**Table 1:** Predictors selected for long-term CVD risk predictions.

Adolescent factors	Variables	Reference
Sociodemographic	Gender Age Race	^26,33^
Socioeconomic	Parental education Parental income Family structure	^32,33^
Lifestyle and health risk	Self-rated health Physical activities Sedentary behaviors Fast-food consumption Eating breakfast Alcohol use Marijuana use Smoking status Obesity Sleep duration Parental obesity Parental diabetes	^32,33,37^
Psychological health	Positive mood Self-image Depressive symptoms Stressful life events	^36,38,40^
Stressful life events	Saw violence Threatened by knife or a gun Was stabbed Was jumped Skipped necessary medical care Suffered a serious injury Was raped Friend attempted suicide Family member attempted suicide Was injured in a physical fight Hurt someone in a physical fight Romantic relationship ended Contacted a STD Run away from home Suffered verbal abuse in romantic relationship	^36,38,40^

Positive well-being factors such as positive mood and self-image are created from participants’ responses to the 10-item Center of Epidemiologic Studies Depression (CES-D) Scale^43^. Four of these items asked about the following feelings experienced in the last week—happiness, feeling as good as other people, enjoying life, and hopefulness. The response to these four questions is summed to generate a single positive mood factor^36,38,39^. The other six questions asked participants whether they– have good qualities, have a lot to be proud of, like themselves, do things right, are socially accepted, and feel loved and wanted. The answers to these questions are added to measure the self-image of each participant^36,40,42^. Adolescent depression is self-reported and measured based on 15 questions from the CES-D questionnaire. The responses to these 15 questions are summed to create a depressive score that ranged from 0 to 45, with 45 indicating higher depression^32,37,44^. The Add Health survey items associated with adolescent predictors (Table 1) are provided in the Supplementary Information.

#### Output variable

The 30-year adulthood CVD risk category (low or high risk) is estimated using Wave IV survey data collected 14 years after the initial interview. In addition, the survey collected information related to participants’ demographics, anthropometric measures, and health test results. We used the risk prediction function, a modified Cox model, derived by Pencina et al., to compute the adulthood CVD risk over a 30-year time frame^24^. The model leverages factors from Wave IV, including—age, gender, systolic blood pressure (SBP), BMI, smoking status, use of antihypertensive medications, and presence of diabetes to estimate the 30-year CVD risk score. Similar to previous research, an individual with a risk score over 20% is classified as high-risk and low-risk otherwise^46^. Thus, the outcome variable is the long-term CVD risk (low or high) of an adolescent.

### ML model development and analysis

The problem of predicting the CVD risk (i.e., categorizing individuals as low and high risk of CVD) is modeled as a supervised classification problem. Recent research has demonstrated the capability of decision trees (DT), random forest (RF), extreme gradient boosting (XGBoost), and deep neural networks (DNN) to accurately predict binary variables in the healthcare domain, such as disease risk^47^, heart disease^48^, and mortality risk^49^. Therefore, we employed and evaluated these four ML models for CVD risk classification. In addition, we considered logistic regression (LR), a traditional multivariate statistical learning model, to benchmark the predictive performance of the four ML models. Stratified random sampling is performed to divide the data into two parts—75% is used for training the classification models, and the remaining 25% is held-out for evaluation. A tenfold cross-validation procedure is employed to eschew overfitting (learning noise) in the learning phase^50^. Furthermore, to calibrate the ML model, its hyperparameters are tuned using the grid-search procedure (see Supplemental Information for detailed procedure)^51^.

### Statistical analysis and model evaluation

The classification models are compared based on three measures, namely, misclassification rate (MCR), area under the receiver operating characteristic curve (AUC-ROC) and area under the precision-recall curve (AUC-PR). The MCR is the percentage of individuals whose CVD risk is incorrectly classified by ML model. Thus, a lower MCR is typically preferred. The McNemar’s test is used to compare the statistical significance of the MCR achieved by two different ML models. The AUC-ROC (or equivalently *c*-statistic) is a single measure for evaluating the overall discriminative performance of the ML model^52^. Besides, AUC-ROC has been consistently used in prior research dealing with classification^53–56^. On the other hand, AUC-PR is considered to be a robust metric for evaluating models dealing with class imbalances^57^. The value for these metrics ranges from 0 to 1, where a higher score indicates good classification capability. The DeLong’s non-parameteric test^58^ and bootstrap-based test^59^ are used to compare the AUC-ROC and AUC-PR of different ML models, respectively. For all the statistical tests, the significance was set at 0.05.

### Mitigating potential biases in predictive modeling

The CVD risk prediction model relies on historical individual-level representative longitudinal data to map a set of predictors to an outcome variable. Given the different number of phases/steps associated with the predictive modeling pipeline, an ML algorithm is vulnerable to several biases that result in skewed/inaccurate predictions^60^. In this research, we have adopted the best strategies suggested in the literature to mitigate the potential biases in the CVD prediction model^61^. The two common biases during data preparation are representation and measurement biases. To mitigate the risk of input data not representing the underlying population (i.e., representation bias), we established inclusion and exclusion criteria (discussed in section “Data description”) to avoid selecting data points that may not be reflective of the population considered in this research. In addition, the variables chosen to measure a specific risk factor (e.g., physical activity, depression) are guided by prior literature evidence and expert opinion, which, in turn, reduces the risk of measurement bias (i.e., choosing an imperfect proxy variable for that risk factor). On the other hand, the ML model training/development phase can introduce algorithmic bias, where the predictions are skewed for certain groups. An algorithmic bias may be caused by improper training data sampling and insufficient training data. To mitigate algorithmic bias, we have employed the following strategies: (i) adopted stratified sampling to prepare the training data (as opposed to random) to ensure a representative number of samples under each sociodemographic category, (ii) employed a weighting scheme to impose a higher penalty for misclassifying a minority class, where the weight for each class is inversely proportional to its frequency in the training data, (iii) developed multiple ML algorithms that adopt different learning methods (e.g., bagging, boosting). Finally, our stratified sampling of data splitting reduces the risk of evaluation bias occurring due to non-representative testing population. Furthermore, we also consider multiple performance metrics (AUC-ROC, AUC-PR and MCR) to mitigate evaluation bias stemming from using improper evaluation metrics.

### ML interpretability and explainability

While RF, XGBoost and DNN have demonstrated high-prediction accuracy than simpler models such as DT in prior studies, they are also regarded as ‘black-box’ models since it is difficult for humans to comprehend their behavior in predicting the outcome. Interpretability of the ML model is the degree to which a model can explain its output based on a set of inputs^62^. The ability to interpret or explain the ML model predictions is crucial for data-driven decision-making, especially for adopting targeted interventions in healthcare. The scope of ML model interpretation can be global or local. Global interpretability identifies how the model makes its predictions based on a holistic view of its features, parameters, and structure. In other words, it explains the global output of the model on an abstract level^63^. On the other hand, local interpretability is achieved by designing more justified model architectures that explain a single prediction^63^.

Existing methods for interpreting ML models can be categorized into two groups—intrinsically interpretable and model-agonistic^64^. The former category of methods is limited to self-explainable models, such as logistic regression and DT models. These models are less complex and easy to explain since a mathematical rule can represent their internal structure. On the other hand, agonistic methods are not restricted and apply to any ML model. In addition, agonistic methods work by analyzing the relationship between the inputs and output rather than analyzing the internal structure as in the intrinsically interpretable methods^64^. In this research, we use Shapley Additive Explanations (SHAP), a model-agonistic approach that adopts the concepts from cooperative game theory^65^. It calculates the contribution of each feature *i* based on the Shapely value^66^, as shown in Eq. (1), where *F* is the entire feature set, and *S* denotes a subset, $S\cup \left\{i\right\}$ is the union of subset *S* and feature *i*, $\left(v\left(S\cup \left\{i\right\}\right)-v\left(S\right)\right)$ is the marginal contribution of feature *i*.

1 $${\varphi }_{i}\left(v\right)=\sum_{S\subseteq F\{i\}}\frac{\left|S\right|!\left(F-\left|S\right|-1\right)!}{F!} \left(v\left(S\cup \left\{i\right\}\right)-v\left(S\right)\right)$$

## Results

The procedure for predicting the CVD risk category using ML algorithms is implemented in Python software on a computer configured with Intel Core i7 3.4 GHz processor, macOS Sierra operating system, and 32 GB RAM. The pre-processed dataset contained 14,083 records, and 75% of it is used for training the ML algorithms while the remaining 25% is used for evaluation. The percentage of high CVD risk subjects in training and testing datasets are 17%, and 16%, respectively.

### Predictive performance of classification models

**Figure 2:**
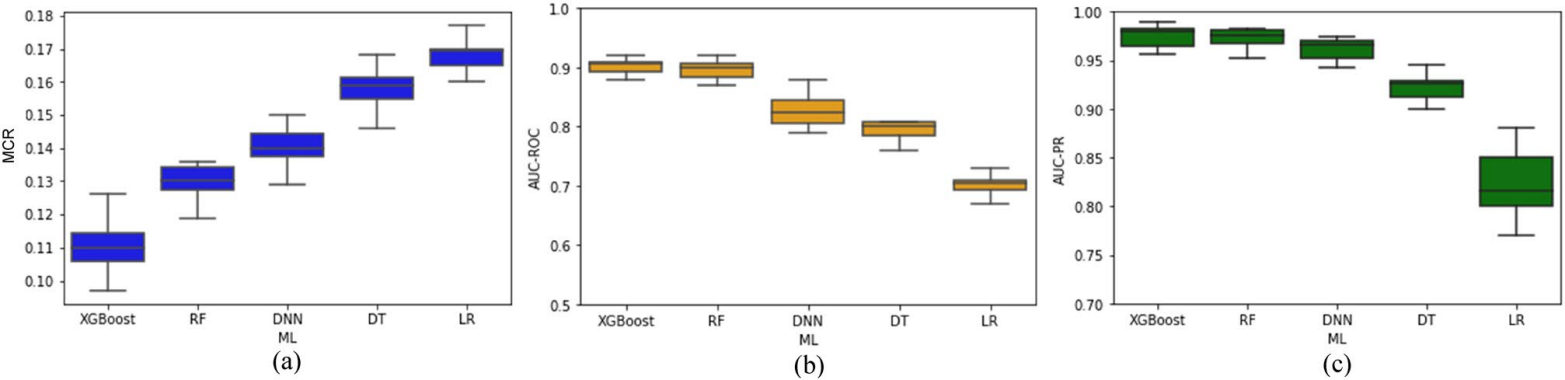
Performance of ML models during tenfold cross-validation procedure.

The performance of classification models on tenfold cross-validation and testing datasets is illustrated in Figs. 2. and 3., respectively. The evaluation metrics indicate a good discriminative capability of “Low” and “High” CVD-risk of all the classification models, except logistic regression. The pairwise McNemar’s test showed that the MCR of LR is significantly worse (*p* < 0.05) than the other ML models under consideration. Besides, the DeLong’s and bootstrap-based tests confirmed that LR had statistically lower AUC-ROC and AUC-PR curves (*p* < 0.05), respectively, than the other ML models. While XGBoost yielded the best average values for all the classification evaluation metrics under consideration, its performance is not significantly different from RF with respect to MCR, AUC-PR or AUC-ROC (*p*-value > 0.05). On the other hand, when compared to DT, XGBoost showed significant improvement with respect to all three performance measures (MCR, AUC-ROC and AUC-PR). Likewise, XGBoost achieved significantly better performance than DNN. The performance of ML models on the testing dataset is comparable to the cross-validation results, thereby suggesting the ML models’ generalization capability. Besides, XGBoost and RF consistently outperformed the other two algorithms for CVD risk classification.

**Figure 3:**
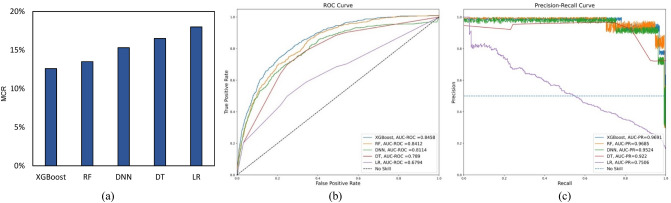
Performance of the ML models on the testing dataset. AUC-ROC curve is maximized in the upper left corner, and AUC-PR curve is maximized in the upper right corner.

### ML model interpretation

This section presents the results associated with ML models’ global and local interpretation. Note that we do not consider the interpretation of the logistic regression model for two reasons—(i) it is a self-explainable model (as discussed in section “Mitigating potential biases in predictive modeling”) and therefore does not require other methods to interpret its predictions, and (ii) it greatly underperformed in predicting the CVD risk (as shown in Figs. 2 and 3) in comparison to the other ML models.

#### Global interpretation of ML models

**Figure 4:**
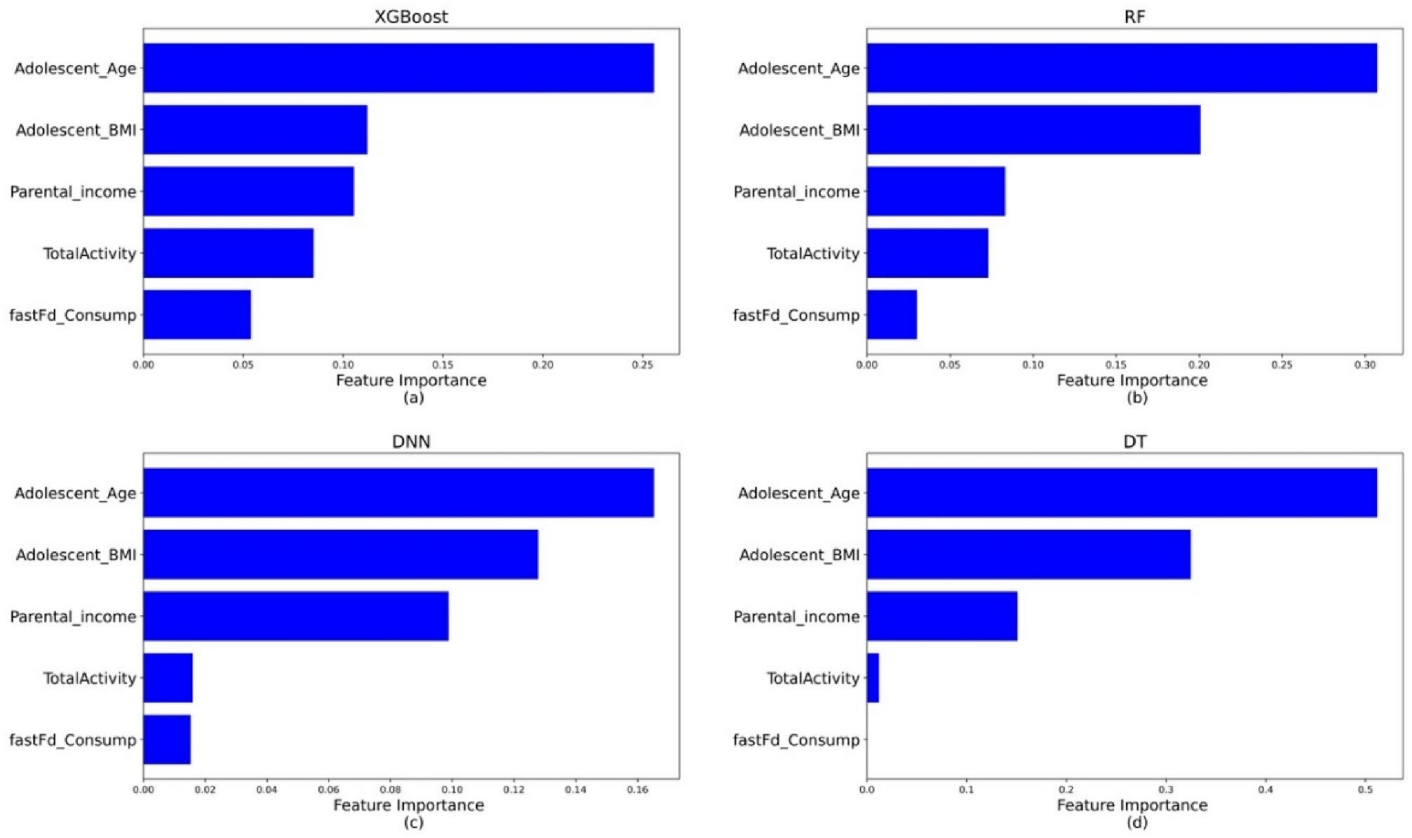
Permutation feature importance plot of the ML models. Higher value corresponds to a more important feature in predicting CVD risk. The plot is created for (**a**) XGBoost, (**b**) RF, (**c**) DNN, (**d**) DT.

**Figure 5:**
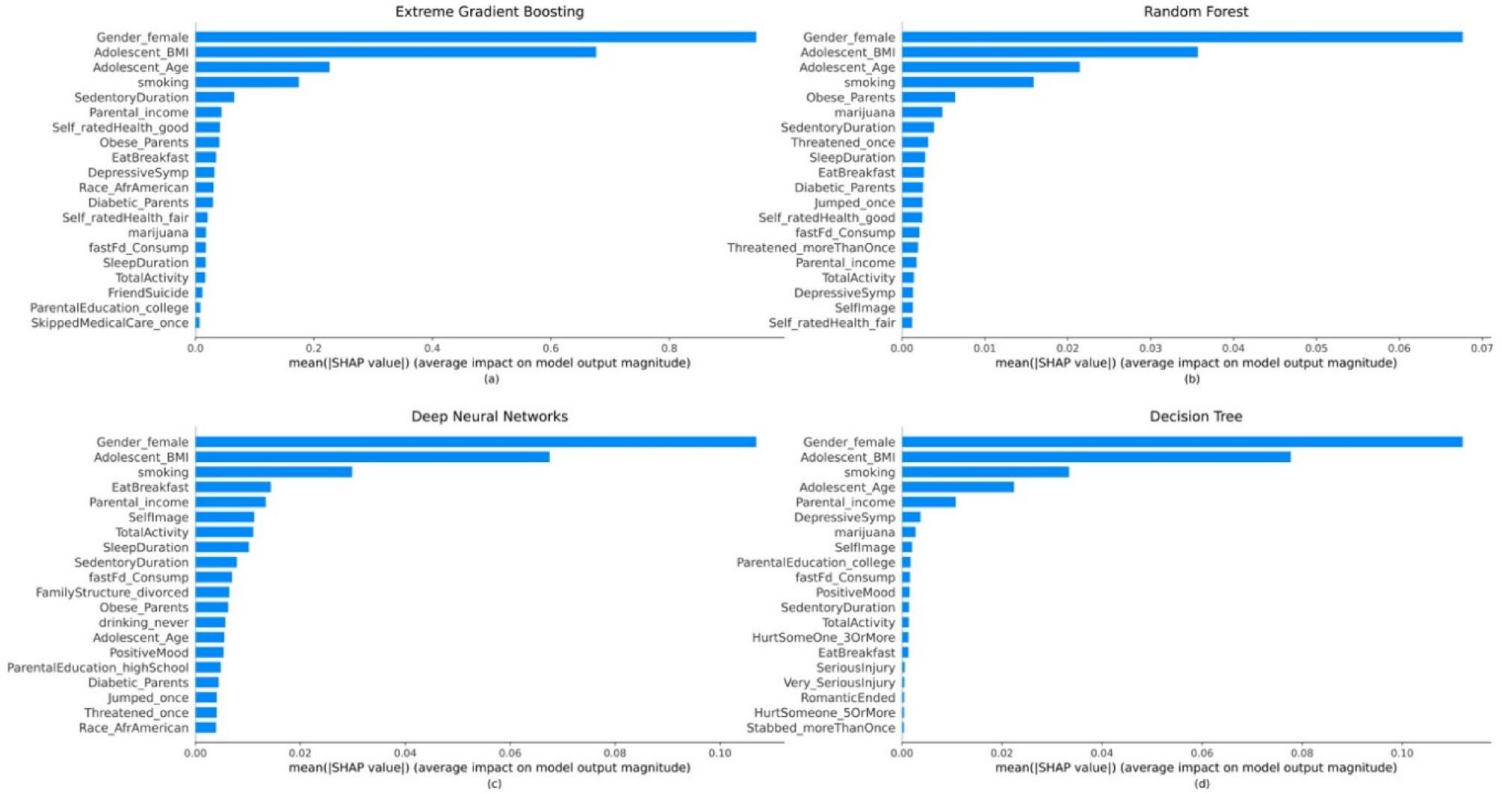
Global interpretation of ML models. The *x*-axis is the average (absolute) SHAP value for each adolescent risk factor. Higher value corresponds to a more important feature in predicting CVD risk. The plot is created for (**a**) XGBoost, (**b**) RF, (**c**) DNN, (**d**) DT.

The global interpretation of the predictions obtained can be interpreted in different ways. The permutation feature importance is shown in Fig. 4, where the predictor’s usefulness is determined by measuring the decrease in classification performance when that variable is not available. On the other hand, Fig. 5. shows the feature importance that is calculated based on the average of absolute shapely values across the entire dataset^66^.

It can be observed from Fig. 4 that the five most important variables for predicting long-term CVD risk are the same, but the degree of importance changes. Moreover, variables corresponding to demographic, socioeconomic, lifestyle and psychological factors are observed to be crucial for predicting the outcome. Alternatively, the global interpretation based on SHAP values suggests Gender and BMI to be the two most important features in all ML models. In addition, smoking status consistently featured as the top predictor for all the models. In the case of RF, besides smoking, using marijuana was an important predictor of high CVD risk. In addition, parental obesity, self-image, eating habits, parental income and depressive symptoms are considered to be important predictors of long-term CVD risk by one or more ML models under consideration.

**Figure 6:**
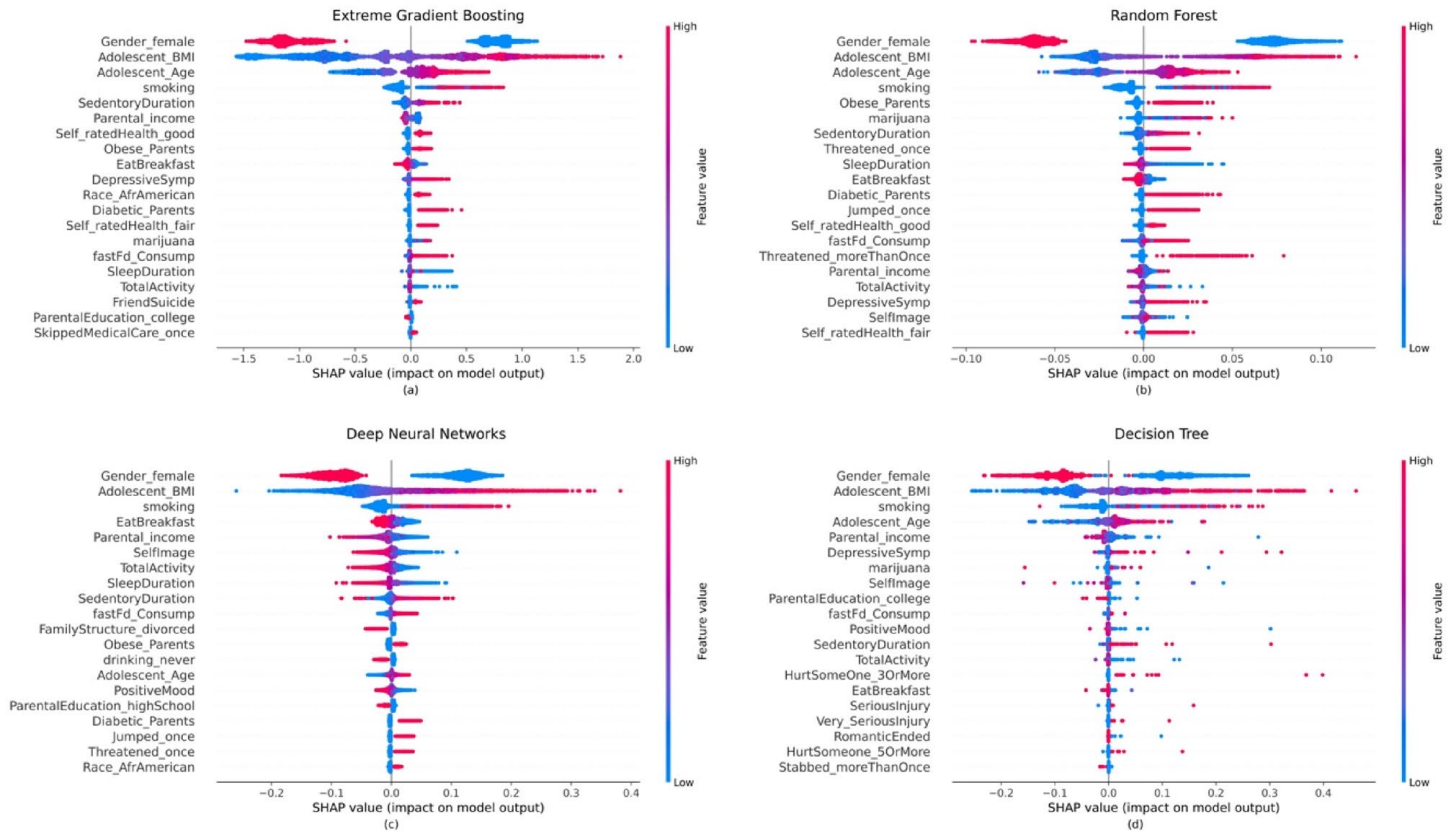
Global interpretation of ML models—SHAP summary plots of the input features. Features were sorted in descending order by SHAP values. SHAP values for each feature were calculated, which is represented by a single dot. Dots were colored based on the underlying feature’s value. For the features of gender_female, the red dots indicated female and the blue dots indicated male. The summary plot is created for each ML model: (**a**) XGBoost, (**b**) RF, (**c**) DNN, (**d**) DT.

As mentioned earlier, the importance plots only show the global influence of each feature on the prediction. However, they do not indicate how each predictor’s contribution positively or negatively affects the prediction. For that reason, summary plots are employed, which provide a global macro-level explanation of how the input variables contribute to the prediction. Figure 6 presents the summary plot demonstrating the importance, impact, original value, and correlation of the adolescent factors to high adulthood CVD risk category. Note that the importance is demonstrated by the decreasing order of the variables. In particular, the impact (positive vs. negative) is shown on the *x*-axis. The color indicates the value of a specific variable, in which red signifies a high value and blue implies a low value. In the case of categorical predictors, the red color indicates the presence of the factor (or true), while blue denotes the value of that variable to be false. For instance, “Gender_female” represents the encoded gender variable where red represents the female gender and blue indicates the male participants. Similarly, “Obese_parents” indicate parents who are obese if the value is “yes” and non-obese otherwise. The correlation of each variable with the target can be inferred when considering both the impact and color of the observations for a specific variable^67^. The importance plots for all ML models show that males are more likely to be in the high CVD risk category as opposed to females. As expected, the likelihood of being categorized as high-risk increases with Age, BMI, and cigarette smoking. On the other hand, the XGBoost (Fig. 5a) shows that less sedentary durations (or being physically active) and higher parental income tend to have a lower influence on the prediction of the high-risk category compared to the low-risk category. For RF, using marijuana and having an obese parent increase the probability of being classified as a high risk. According to DNN plots, having a low self-image and less occurrence of eating breakfast increase the likelihood of being classified as a high-risk. The higher the depressive symptoms, the higher the chance of being classified as high-risk according to DT.

#### SHAP dependence plot: global interpretability

**Figure 7:**
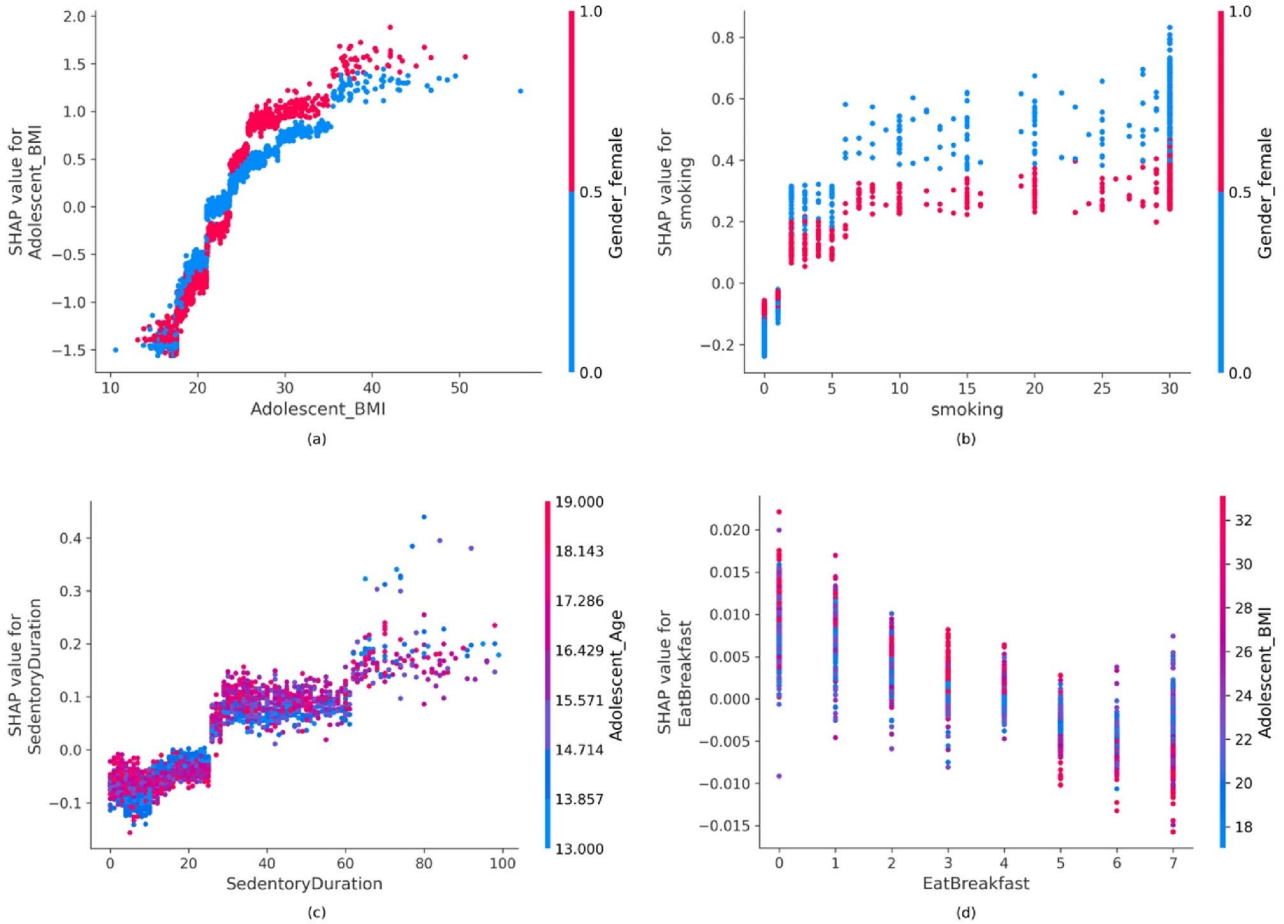
Partial dependence plots: (**a**) adolescent BMI, (**b**) cigarettes smoked per month, (**c**) hours of sedentary duration, (**d**) breakfast frequency. SHAP values greter than zero indicates a positive correlation between the two adolescent risk factors.

Other than the demographic variables (i.e., age and gender), some lifestyle and health risk variables that appear to affect the risk of CVD are BMI, smoking, sedentary duration, and weekly breakfast frequency. To show these features’ marginal effect on the ML models’ outcome, dependence plots are used (Fig. 7). These plots view the relationship between the feature and the feature’s impact on the model. In addition, it includes another variable for coloring (red or blue) to highlight possible interactions. If the SHAP values increase with the increasing values of the feature, then it would indicate a positive correlation between the feature and the predicted outcome; otherwise, it would signify a negative relationship.

For instance, Fig. 7a shows an approximately positive and linear relationship between BMI and the high CVD risk category, and that BMI interacts mainly with gender. Similarly, as the instance of smoking increases, this also increases CVD risk, as shown in Fig. 7b. The figure also shows that males (in blue) who smoke more than two cigarettes a month have a higher risk of CVD than females (in red) who also smoke the same number of cigarettes. Sedentary durations seem to interact mainly with age, as shown in Fig. 7c. It also can be seen that individuals who are 15 years and older and have more than 24 h of inactive durations a week have a positive and approximately linear relationship with higher CVD risk. Figure 7d illustrates that eating breakfast more often decreases CVD risk. It also shows that individuals who eat breakfast more than once a week have a lower BMI than those who have breakfast once a week or do not have it.

#### Individual SHAP value plot: local interpretability

**Figure 8:**
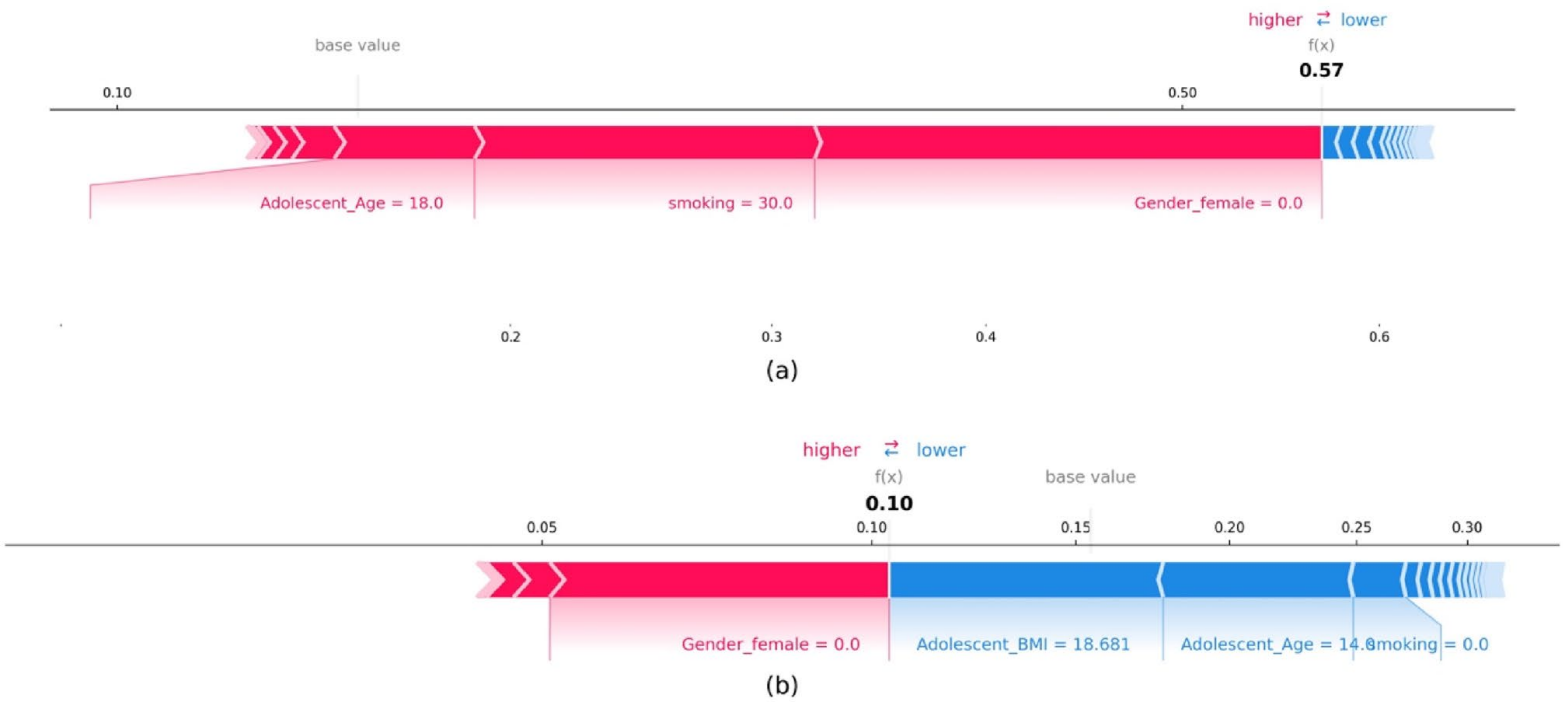
Local interpretation—force plots for two individuals from the testing set of the XGBoost model: (**a**) high risk individual, (**b**) low risk individual.

In addition to the global interpretation of the entire dataset, SHAP provides local interpretation for each sample. The individual plot, as shown in Fig. 8a and b, illustrate the classification of two samples as high risk and low risk, respectively. The individual/force plot shows how each feature influences the classification of each observation as high or low risk, as well as the direction and magnitude of the influence. In the context of classification, the red color represents features that drive the classification to be in the high-risk category, while the blue color shows those nudging the prediction to be in the low-risk category. The length of the bar denotes the magnitude of influence for the corresponding feature. For instance, features with a more extended bar indicate having more influence on the output^67^. The bold value is the probability of the output being predicted as a particular risk category. A higher probability than the cut-off (in our case, the cut-off is kept at the default setting of 0.5) leads the model to classify it as a high risk, and low risk otherwise ^68^. For instance, the selected individual in Fig. 8a is a male who is 18 years old and has smoked every day for the past 30 days. The individual plot explains how the model perceives this individual. It can be seen from the figure that the predictors, Age, Smoking, and Gender, are pushing the model to classify it as a high risk. It can also be seen that Gender has a larger influence, followed by Smoking and Age for this individual. On the other hand, Fig. 8b shows a male individual who is 14 years old, has a normal BMI and does not smoke. In this case, the BMI, Age, and Smoking status push the model to classify it as a low risk, whereas the male gender pushes it to be classified as a high risk. The combined influence of normal BMI, younger age, and non-smoking leads to a prediction of 0.10, which is the probability of being classified as high-risk. Since this is less than the threshold of 0.5, the predicted risk category is low-risk.

## Discussion

This study provides the first long-term ML-based CVD risk prediction model among adolescents based on a longitudinal dataset. We trained four ML models, namely XGBoost, RF, DDNs, and DT, to predict CVD risk, using 36 predictors, and compared the model performances measured by MCR, AUC-ROC and AUC-PR. The results of the prediction models indicated that adolescent risk factors were able to predict the CVD risk with high accuracy. This finding supports the prior research highlighting the importance of adolescent risk factors in developing CVD events later in life^9–11^. Moreover, our results suggest ML models to be capable of accurately predicting long-term risk of CVD among adolescents. This finding complements other research works that have employed ML to predict the long-term risk of other diseases such as type 2 diabetes^69^, kidney allograft survival^70^, cancer^71^.

This study also identified adolescents’ risk factors that were important for predicting long-term CVD risk. Consistent with previous research, our findings reveal that Gender, Age, BMI, and smoking were important predictors of CVD risk^12,21^. Earlier studies have established an association between parental income^72^, sedentary duration^73^, skipping breakfast^74^, self-image^75^, and depressive symptoms^76^ and a higher likelihood of CVD risk, and our study results further substantiated this as these factors are found to be critical predictors of CVD risk. In addition, some predictors emerged as important for specific algorithms but not for others. This could be due to the learning pattern of the ML algorithm and the way they select and rank features^77^. For instance, “FamilyStructure_divorced” was ranked as one of the key predictors for CVD by DNN as opposed to the other ML models. Also, stressful life events appeared to have little to no influence on CVD risk, as they were ranked low by all ML models.

Most prior works focusing on interpretability use algorithms such as regression and decision trees, whereas studies focusing on achieving higher prediction accuracy use black-box ML models and compromise interpretability. This research is among the first to provide the local and global interpretation uncovered by the black-box ML model for predicting adulthood CVD risk using adolescent risk factors. For instance, dependence plots revealed how risk factors such as weekly breakfast frequency and BMI interact with each other. Our results indicate that adolescents who eat breakfast more than once a week have a lower BMI than those who eat it once a week or skip it. This finding support prior work highlighting the association between high BMI and skipping breakfast for adolescents^78^.

The findings of this study have several implications. Once the proposed tool is validated on new data sources, it can be used to develop primordial prevention plans that promote youth health and enable individuals to seek care at an early stage. Developing such plans could improve the quality of life, and avoid psychological stress, functional impairment, medication-related side effects, and premature death^79^. Moreover, early intervention could reduce healthcare costs by up to 70%^80^. Therefore, standardizing the proposed approach for other diseases and adapting it to intervene at an earlier stage could achieve substantial cost savings. In addition, the proposed method can be scaled to prevent and manage other diseases such as type 2 diabetes, obesity, and arthritis, thereby improving the overall population health.

Although this study has many merits, it has a few limitations that can guide future research. First, the ML models in this study use the data collected as part of the Add Health study. While the study uses a representative sample of adolescents in the US, the generalization of the proposed ML models for other adolescent cohorts is not evaluated and could be considered as a future research direction. In addition, the capability of our ML models to predict CVD risk for individuals who may fall outside the age ranges considered in this study is not known. Second, the impact of certain predictors such as adolescent waist circumference, heart rate and family history of CVD was not considered as it was not collected as part of the Add Health study. Third, this research does not seek to optimize or reduce the number of features but instead uses all available risk factors that had demonstrated significant association with CVD risk in prior studies. While the predictive performance is less likely to be skewed due to our approach, future work could consider optimizing the predictors through recursive feature elimination to develop a parsimonious ML model. Finally, this research focused on developing and validating an explainable long-term CVD risk prediction model using Add Health data, but aside from handling the black-box nature, there are numerous other aspects (such as data shift and external validation) to be considered for the safe translation of such predictive models into clinical settings. More specifically, the outcome variable employed in this study was collected 14 years ago (2007–2008) from Wave IV of the Add Health study, therefore, performance of the ML model for more recent data needs to be assessed and compared with the results reported in this research. Likewise, the model performance could not be evaluated on an external validated cohort (i.e., a data source that is not part of the Add Health study) since it was not possible to derive the dataset for other similar longitudinal studies. Potential future work is to establish a retrospective 20-year longitudinal data from electronic medical records of one or more hospitals to validate the performance and generalizability of the ML-based CVD risk prediction model on a new population.

## Conclusions

Although the risk factors leading to adulthood CVD begins early in life, there is currently no tool available to predict the long-term CVD risk among adolescents. In this proof-of-concept study, we demonstrated the capability of ML models to predict the long-term CVD risk of adolescents accurately based on adolescent risk factors. Besides, critical predictors of long-term CVD risk and its impact on risk prediction are obtained using the SHAP approach, an explainable technique for interpreting ML predictions. Successful validation of the proposed framework on other large cohorts can lead to the clinical adoption of an ML-based risk calculator for long-term CVD prediction and facilitate early detection and prevention opportunities.

## Data Availability

The data that support the findings of this study are available from Add Health (http://www.cpc.unc.edu/addhealth) but restrictions apply to the availability of these data, which were used under license for the current study, and so are not publicly available. Data are however available from the authors upon reasonable request and with permission of Add Health.

## Code availability

The code used to analyze the data is available from the corresponding author upon request.
